# Comparison of Procalcitonin Assays on KRYPTOR and LIAISON^®^ XL Analyzers

**DOI:** 10.3390/diagnostics9030094

**Published:** 2019-08-08

**Authors:** Mariella Dipalo, Cecilia Gnocchi, Paola Avanzini, Roberta Musa, Martina Di Pietro, Rosalia Aloe

**Affiliations:** SSD Biochimica ad Elevata Automazione, Azienda Ospedaliera Universitaria di Parma, Via Gramsci 14, 43126 Parma, Italy

**Keywords:** sepsis, PCT, procalcitonin, immunoassay, antibiotic, chemiluminescence, immunofluorescence

## Abstract

Our laboratory performs procalcitonin (PCT) assays on a Brahms KRYPTOR analyzer with the Brahms PCT sensitive Kryptor kit. In this study, we wanted to compare the assays obtained in this way with the ones performed on the LIAISON^®^ XL. From January to May 2017, 171 samples were analyzed, of which 65 from female patients (age: 22–98 years) and 106 from male patients (age: 16–97 years). The PCT determination was performed using the LIAISON^®^ XL and KRYPTOR analyzers, by chemiluminescence (Chemiluminescence immunoassay—CLIA) (LIAISON^®^ BRAHMS PCT^®^ II GEN) and immunofluorescence (Brahms PCT sensitive Kryptor) assay, respectively. For the LIAISON^®^ BRAHMS PCT^®^ II GEN, 52% of the results were placed between 0.0 and 0.5 ng/mL, 18% between 0.5 and 2.0 ng/mL, and 30% between 2.0 and 100 ng/mL; the mean was 4.09 ng/mL, the median 0.456 ng/mL, the maximum value 97.2 ng/mL, and the minimum value 0.02 ng/mL. For the Brahms PCT sensitive Kryptor, 55% of the results were positioned between 0.0 and 0.5 ng/mL, 21% between 0.5 and 2.0 ng/mL, and 24% between 2.0 and 100 ng/mL; the mean was 3.72 ng/mL, the median 0.39 ng/mL, the maximum value 103 ng/mL, and the minimum value 0.01 ng/mL. The mean of the results obtained with the two methods showed no significant differences (3.717 for Kryptor and 4.094 for LIAISON^®^). PCT assay with Brahms reagents, both on the Kryptor and LIAISON^®^XL platforms, offers excellent performance in terms of sensitivity and specificity.

## 1. Introduction

Sepsis and its consequences are an important cause of mortality and morbidity, both for inpatients and outpatients, in all age groups. Despite the progress made by medicine, diagnosing sepsis today still constitutes a challenge. This is mainly due to the wide variability of the clinical presentation (which, in turn, depends on the site of the initial infectious outbreak), the involved microorganism, the timing of evaluation and clinical intervention, and, finally, the presence of comorbidities and concomitant therapies [1]. Until a few years ago, the C-reactive protein (PCR) assay was the only one that could help with the monitoring of the progress of inflammation, even if in a non-specific way. Blood culture represents the gold standard test for the correct identification of pathogenic species responsible for sepsis, but its execution requires a timeframe that ranges from 24 to 72 h, depending on the complexity of the investigations needed. In recent years, the requests for the determination of procalcitonin (PCT) have been rapidly increasing, also because of the availability of this assay on various analytical platforms using different technologies [2].

Procalcitonin (PCT) is a peptide formed by 116 amino acids (aa), normally synthesized in the C cells of the thyroid, from pre-procalcitonin (composed of 141 aa), which is then converted, once in the blood stream, into a mature form, called calcitonin (composed of 32 aa), involved in the homeostasis of calcium. In patients with severe bacterial infections, PCT synthesis can also occur in other districts (especially in the liver, kidneys, lungs, pancreas, intestine, and leukocytes) due to specific inflammatory stimuli, mainly mediated by interleukin 6 (IL-6) and tumor necrosis factor alpha, which are triggered by the lipopolysaccharide, the major component of the external membrane of Gram-negative bacteria. Under such conditions, the PCT concentration can increase up to 10,000 times compared to the usually normal values, which are very low at below 0.05 ng/mL [3]. This peculiar biological behavior can be used to diagnose serious infections, especially sepsis [4]. The number of studies and meta-analyses evaluating the diagnostic performance of PCT for both the diagnosis and management of sepsis has increased exponentially over the past decade. According to a recent meta-analysis published by Tan et al. [5], PCT shows a diagnostic accuracy of 85% for sepsis (with 0.80 sensitivity and 0.77 specificity), thus presenting a significantly higher precision than the one of C-reactive protein, which stands instead at 73% (with 0.80 sensitivity and 0.61 specificity). More importantly, in another meta-analysis published by Meier et al. [6], the management of antibiotic therapy by means of PCT determination was significantly effective in shortening the duration of the therapy itself (mean change, −2.86 days), thus representing a valuable step towards reducing antibiotic resistance [7]. In general, patients for whom PCT monitoring is required are as follows:

- patients undergoing antibiotic therapy, in order to check the effectiveness of the treatment;
- patients at high risk of severe infections due to the presence of concomitant diseases,
- patients subject to long-term ventilation;
- patients at risk of catheter-related infections;
- patients subject to immunosuppression (oncology, transplant, chemotherapy, neutropenia);
- post-operative or post-traumatic patients;
- patients at high risk of super infections (burns).

The implementation of this test could therefore be of help not only for the early diagnosis of sepsis, but also for the reduction of the risk of antibiotic resistance through targeted therapies, and for the administration of reduced doses of drug, resulting in benefits on health balance in terms of economic savings.

In 2018, our laboratory received 16,083 requests for PCT concentration determination (Table 1), requests that, compared to the previous year, increased by about 15% (total PCT 2017: 13,901). Our laboratory performs PCT assays on a Brahms KRYPTOR analyzer with the Brahms PCT sensitive Kryptor kit. In this study, we wanted to compare the assays obtained in this way with the ones performed on a LIAISON^®^ XL so that we could use the LIAISON^®^ XL instrumentation, in view of a highly automated organization. Moreover, given the importance of using PCT as a tool to monitor antibiotic treatment efficacy, it is important to have diagnostic systems and tests within the same hospital structure that give comparable results because they have the same calibration so that they can be used interchangeably.

## 2. Materials and Methods

From January to May 2017, we processed serum samples from 65 women (age: 22–98 years) and 106 men (age: 16–97 years), for a total of 171 subjects. The study was performed on leftover samples that were completely anonymized and de-identified, thus no informed consent was required. For the analysis, we used the LIAISON^®^ XL analyzer (DiaSorin SpA, Saluggia VC, Italy), with LIAISON^®^ BRAHMS PCT^®^ II GEN reagents and calibrators, and the Brahms KRYPTOR analyzer (Brahms, Hennigsdorf, Germany distributed in Italy by Dasit), with Brahms PCT sensitive Kryptor reagents and calibrators (code M8410000, code MG611PCTK).

**Figure 1:**
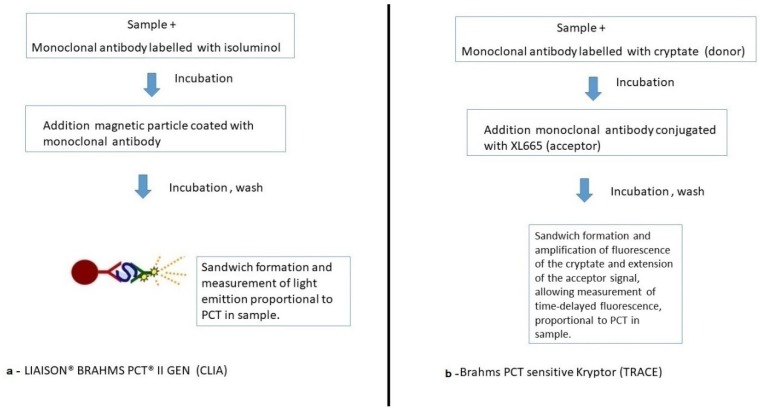
(**a**) Liaison Brahms PCT II Gen and (**b**) Brahms PCT sensitive Kryptor immunofluorescence test mechanism.

The LIAISON^®^ BRAHMS PCT^®^ II GEN test is a sandwich immunoassay based on the principle of chemiluminescence (CLIA), in which magnetic particles (solid phase) coated with a specific monoclonal antibody and another monoclonal antibody (specific for a different epitope of procalcitonin) labeled with an isoluminol derivative are used. During the first incubation, PCT binds to the conjugated antibody. Then, the solid phase is added to the reaction; the sandwich is formed only in the presence of PCT molecules, as they are bound to both antibodies. After the second incubation, the unbound material is removed with a wash cycle. Light emission generated by the chemiluminescent reaction is measured by a photomultiplier as a relative light unit (RLU). The production of light is directly proportional to the concentration of PCT present in the sample (Figure 1a) [8].

The Brahms PCT sensitive Kryptor immunofluorescence test is instead based on the TRACE (time-resolved amplified cryptate emission) technology, which measures the signal emitted by an immune complex at a delayed time.

The TRACE technology is based on the transfer of non-radioactive energy from a donor (cage structure with a europium ion in the center (cryptate)) to an acceptor, which is part of a chemically modified photo-receptive algal protein (XL665). Both the cryptate and XL665 are conjugated with monoclonal antibodies targeted to different epitopes on the PCT molecule.

The proximity between the donor (cryptate) and the acceptor (XL665), when they are part of an immune complex, and the overlap between the emission spectrum of the donor and the absorption spectrum of the acceptor intensify the fluorescence signal of the cryptate, and also extend the duration of the acceptor signal. This allows measurement of the time-delayed fluorescence, which is proportional to the concentration of the analyte to be measured (Figure 1b) [9].

It should be noted that the analysis kits on both platforms allow for the use of the same monoclonal antibodies (solid phase antibodies and antibodies conjugated to XL665, respectively, in the LIAISON^®^ BRAHMS PCT^®^ II GEN method and the Brahms PCT sensitive Kryptor method), targeted against katacalcin, the C-terminal region of procalcitonin, which is formed by 21 amino acids). For the characteristics of reagents, see Table 2.

## 3. Results

With the LIAISON^®^ BRAHMS PCT^®^ II GEN method, 52% of the results were between 0.0 and 0.5 ng/mL, 18% between 0.5 and 2.0 ng/mL, and 30% between 2.0 and 100 ng/mL, and the maximum and minimum values were 97.2 and 0.02 ng/mL, respectively. The mean and median values were 4.09 ng/mL and 0.456 ng/mL, respectively.

With the Brahms PCT sensitive Kryptor method instead, 55% of the results were between 0.0 and 0.5 ng/mL, 21% between 0.5 and 2.0 ng/mL, and 24% between 2.0 and 100 ng/mL, and the maximum and minimum values were 103 ng/mL and 0.01 ng/mL, respectively. The mean and median values were 3.72 and 0.39 ng/mL, respectively.

**Figure 2:**
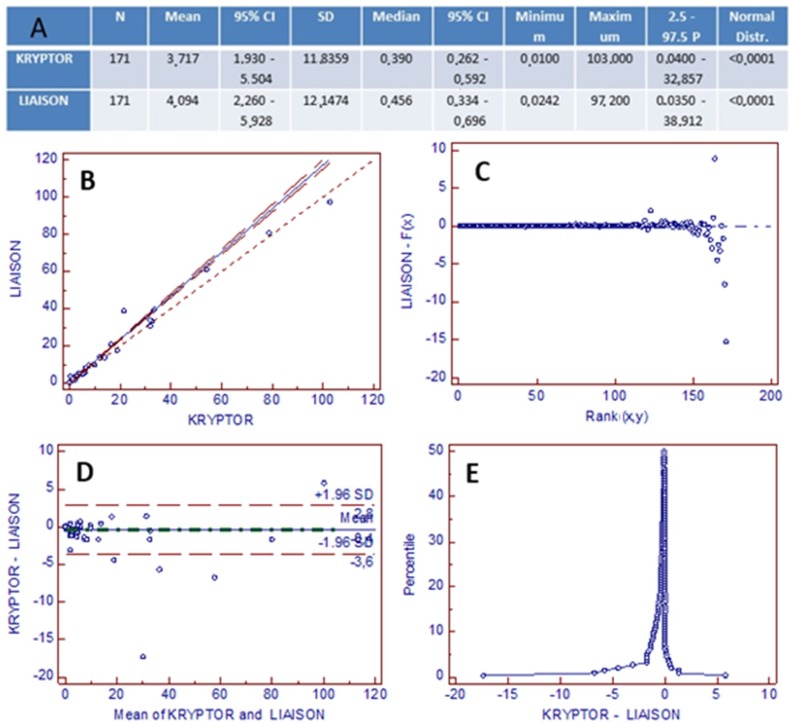
Analysis results. (**A**) Summary table of our data: mean (95% CI), median (95% CI), minimum and maximum of the two methods; (**B**) Linear regression line independent of the sample distribution Passing–Bablok (PB): *y* = −0.0102086 + 1.172143*x* (Pearson coefficient = 0.99); (**C**) PB residual chart; (**D**) Comparison of Bland–Altman graph with representation of the difference, in terms of absolute value, between the two measurements shown according to the mean of the measurements; (**E**) Mountain plot graph or representation of the empirically folded cumulative distribution, obtained by calculating the distribution of percentiles relative to the differences between the two methods placed in an orderly manner.

There are no significant differences between the mean and the median of the results obtained with the two methods (mean 3.717 for Kryptor and 4.094 for LIAISON^®^; median 0.39 for Kryptor and 0.45 for LIAISON^®^), with the distribution of values following normality (Normal distribution: <0.001) (Figure 2A).

The analysis shows an excellent correlation between the results obtained on the LIAISON^®^XL analyzer and on the Brahms Kryptor analyzer, with the respective reagents, both in terms of slope and intercept (Pearson coefficient: 0.99) (Figure 2B).

The regression model is further validated by the residual analysis (Figure 2C).

The Bland–Altman graph obtained by comparing the measurement differences between the two methods as a function of the average of the measurements shows a reduced dispersion of values around the mean and within the standard deviations, without significant differences (Figure 2D). The absence of significant differences is also reflected in the mountain plot graph (Figure 2E), where the graph is in fact centered on zero, missing values along the two tails.

In the literature, there is growing evidence about the fact that the determination of PCT in the monitoring of antibiotic therapy is significantly useful [6]. In this context, the PCT assay, once the appropriate antibiotic therapy has been set, allows the clinician to highlight the patient’s effective response to the antibiotic, thus reducing the risk of unnecessarily prolonged treatments, the development of resistance, and other side effects.

In this regard, we have followed the course of PCT concentrations in some patients undergoing antibiotic therapy over time.

These patients, coming respectively from the emergency medicine department (patient 1, man 78 years old), a long-term care setting (patient 2, man 83 years old), and medical oncology (patient 3, woman 55 years old), were part of the population used for comparing the two methods.

The PCT determination was performed with the two assay methods throughout the antibiotic administration period, obtaining the following results.

**Figure 3:**
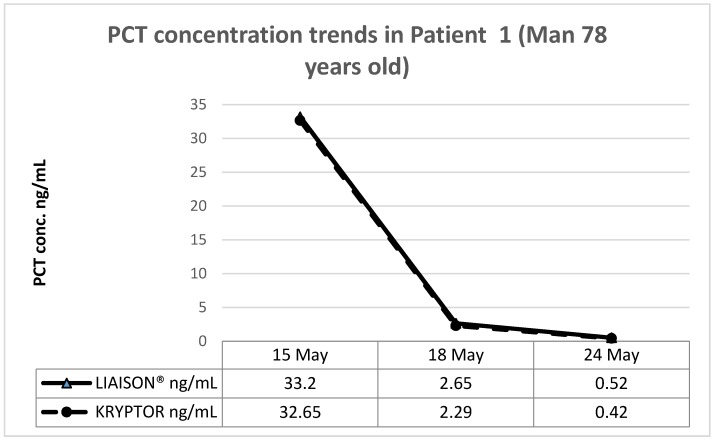
Patient 1 (Internal medicine, man 78 years old).

**Figure 4:**
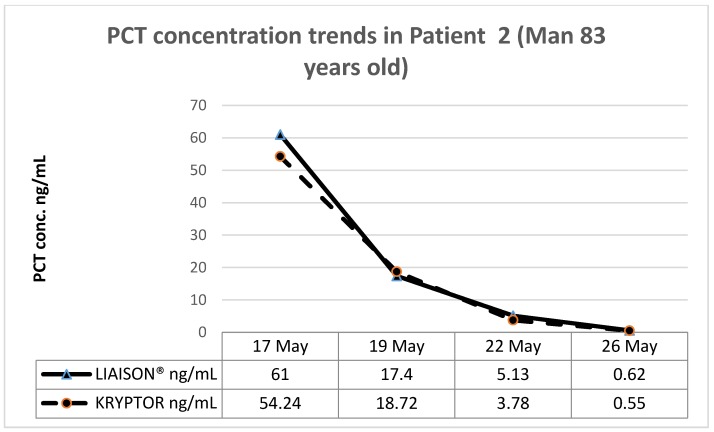
Patient 2 (Long-term hospitalization, man 83 years old).

**Figure 5:**
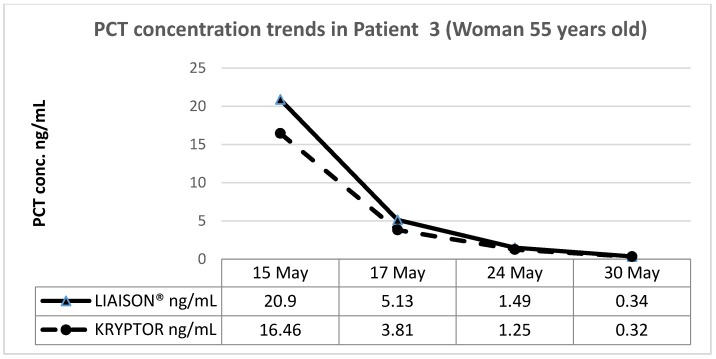
Patient 3 (Medical oncology, woman 55 years old).

Figure 3, Figure 4 and Figure 5 show the trend of PCT concentrations in the three patients (PCT LIAISON^®^ blue line; Kryptor orange line). In all three cases, the PCT values measured with LIAISON^®^ and Kryptor are completely similar to each other, as shown by the overlap of the graphs. Furthermore, it is possible to observe a clear decrease in PCT values over time, in response to antibiotic therapy.

## 4. Discussion

The determination of PCT with Brahms reagents, both on the Kryptor and LIAISON^®^XL platforms, presents excellent performance in terms of sensitivity and specificity. The results obtained show a high correlation between the assays performed on the two analyzers. However, the current lack of standardization of the different methods available to measure PCT remains an unmet target. Currently, PCT is determined by enzymatic, luminometric, chemiluminescent, electrochemiluminescent, fluorescent, and turbidimetric immunoassays, and the latter can be adapted for use on a large number of analytical clinical chemistry platforms, thus favoring the widespread availability of the test.

The LIAISON^®^ BRAHMS PCT^®^ II GEN method could be a key component for PCT-monitored antibiotic therapy and for the initial diagnosis of bacterial infection, offering good quality as well as accurate and acceptable PCT measurements. However, as with other commercially available PCT determination tests, the results must be interpreted carefully in the context of medical history, physical examination, and microbiological evaluation, given that the increase in PCT levels are not always correlated with infection and that low levels of PCT do not automatically exclude the presence of bacterial infection [10]. Therefore, the test results should not replace clinical judgment but should be integrated in a broader context, in order to obtain a better diagnostic performance [11].

## Figures and Tables

**Table 1:** Departments that requested procalcitonin (PCT).

PCT Requests by Department		
Departments	PCT Requests	%
Long-term Hospitalization	3756	23%
Intensive Care Unit	1906	12%
Medical Immunology Department	1394	9%
Internal Medicine	1272	8%
Surgery	1003	6%
Medical Therapy Department	975	6%
Cardiology	923	6%
Hematology	908	6%
Pneumology	680	4%
Emergency Medicine–Emergency Room (ER)	675	4%
Orthopedy	630	4%
Nephrology	459	3%
Medical Oncology	408	3%
Others	397	2%
Urology	283	2%
Transplants	263	2%
Neurology	151	1%

**Table 2:** Characteristics of used reagents.

Characteristics of Used Reagents		
	LIAISON^®^ BRAHMS PCT^®^ GEN	Brahms PCT Sensitive Kryptor
Number of tests	100	100
Principle	sandwich 1 step	sandwich 1 step
Signal	Isoluminol	TRACE (time-resolved amplified cryptate emission)
Specimen	Serum, plasma	Serum, plasma
Volume of sample	100	50
Frequency of calibration	56 days	7 days
Functional Sensitivity or Limit of Quantitation (LoQ)	0.04 ng/mL	0.06 ng/mL
Limit of Detection (LoD)	0.02 ng/mL	0.02 ng/mL
Limit of Blank (LoB)		
Measuring Range ng/mL	0.02–100	0.02–50 ng/mL
Sample Dilution	Automatic	Automatic
Calibrator and Integral Stability	12 weeks	14 days
Time to first results	16 min	19 min

Regarding the statistical analysis, MedCalc 18.6 software (MedCalc bvba software, Ostend, Belgium) was used.
